# A Case Report on Laryngeal Histoplasmosis Masquerading As Tuberculosis

**DOI:** 10.7759/cureus.79171

**Published:** 2025-02-17

**Authors:** Naznin Naher, Sunil K Biswas, Hasan Imam, Md Nazmul Hasan

**Affiliations:** 1 Department of Internal Medicine, Bangabandhu Sheikh Mujib Medical University, Dhaka, BGD

**Keywords:** dimorphic fungus, fiber optic laryngoscopy, fungal stain, hoarseness of voice, laryngeal histoplasmosis

## Abstract

Laryngeal histoplasmosis is a rare manifestation of infection by Histoplasma capsulatum, typically resulting from hematogenous dissemination following a primary pulmonary infection. However, primary laryngeal involvement without prior lung disease is uncommon and can present a diagnostic challenge. The disease often mimics laryngeal malignancies or other chronic inflammatory conditions due to its nonspecific symptoms, including progressive hoarseness, dysphagia, odynophagia, fatigue, malaise, and weight loss. Risk factors include immunosuppression, such as HIV/AIDS, prolonged corticosteroid use, or other conditions leading to impaired cellular immunity. Diagnosis requires a high index of suspicion, particularly in endemic regions, and is confirmed through histopathological examination, fungal cultures, or molecular testing of laryngeal biopsy specimens. Due to its rarity, misdiagnosis is common, leading to delays in appropriate antifungal therapy, which primarily consists of itraconazole or amphotericin B in severe cases. Early recognition and treatment are crucial to prevent complications and improve patient outcomes. This report highlights the need for increased awareness of laryngeal histoplasmosis among clinicians to facilitate timely diagnosis and management. We report the case of a 25-year-old male presented with dysphagia and increasing hoarseness of voice over three months, initially suspected to have tuberculosis (TB) but later diagnosed with primary laryngeal histoplasmosis.

## Introduction

Histoplasmosis is a non-contagious fungal infection caused by Histoplasma capsulatum, which thrives in warm, humid environments, especially in soil enriched with bird and bat droppings. It is most common in North America, with fewer cases in Asia and Europe. Inhalation of the microconidia of fungus leads to lung infection, where it converts to its yeast form and can spread throughout the body via the bloodstream and lymphatic system. Histoplasmosis was first described in 1906 by a pathologist named Samuel Taylor Darling. Initially, the organism was discovered while a patient was being autopsied. In 1906, he made the first identification and description of the organism based on postmortem investigations of the adrenal glands, lungs, liver, spleen, and lymph nodes. Dodd and Tompkins discovered histoplasmosis in a live infant in 1934 [1]. The first histoplasmosis survey was done in Bangladesh in 1961 (then East Pakistan), which revealed that 12-23% of people had a positive skin reaction to histoplasmin [2]. Similar findings were also discovered in a 1960 survey conducted among residents along the Jamuna River's banks in the vicinity of Delhi, India. More than half of the population in endemic areas has a positive skin reaction to histoplasmin [3]. The first case of histoplasmosis was reported in Bangladesh in 1982 and the second case was detected in 2005 [4]. The clinical symptoms of histoplasmosis are similar to those of laryngeal cancer or tuberculosis, making it sometimes difficult for many clinicians to diagnose the disease [5]. 

## Case presentation

A 25-year-old male presented with a three-month history of progressive hoarseness and dysphagia, primarily due to pain during swallowing. He also reported intermittent fever, cough, and significant weight loss over the past month. He has a history of post-tuberculosis (TB) fibrosis following two previous episodes of pulmonary TB in 2002 and 2017, both of which were successfully treated. Additionally, he has chronic calcific pancreatitis, for which he is receiving pancreatic enzyme supplements and dietary modifications, and was recently diagnosed with diabetes mellitus. The patient is a smoker and does not use alcohol or illicit drugs. Furthermore, he reported no exposure to caves or bats. He had no stridor or signs of respiratory distress but was noted to have hoarseness of voice. The patient exhibited mild anemia, with no cervical lymphadenopathy or hepatosplenomegaly, but coarse crepitations were heard in the lungs, suggesting disseminated TB as a provisional diagnosis involving both the lungs and larynx.

TB was suspected first over laryngeal carcinoma and histoplasmosis because it is endemic in our region, and the patient had a history of two prior TB episodes, which increases the risk of recurrence. Carcinoma was less likely due to the patient's young age, and histoplasmosis was considered unlikely due to the lack of exposure to caves or bats, which are common risk factors for the disease. Blood tests showed low hemoglobin, high ESR, and normocytic normochromic anemia (Table 1). Sputum tests, including fungal stain and GeneXpert, were negative, and sputum culture grew normal flora (Table 2). HIV, hepatitis B, and C panels were all normal (Table 2).

**Table 1:** Complete blood count during admission. CBC, complete blood count; WBC, white blood cells; RBC, red blood cells; MCV, mean corpuscular volume; MCH, mean corpuscular hemoglobin; ESR, erythrocyte sedimentation rate; M, male; Hb, hemoglobin; Ht, hematocrit

Parameter	Patient value	Reference range
Hb	10 g/dL	13.5-17.5 g/dL (M)
Ht	35%	41-50% (M)
MCV	88 fL	81.0-97.0 fL
MCH	30 pg	26-32 pg
RBC count	4.12×10¹²/L (M)	4.7-6.1×10¹²/L (M)
Total leukocytes (WBCs)	10×10⁹/L	4.5-11×10⁹/L
Neutrophils	70%	40-70%
Lymphocytes	20%	20-40%
Monocyte	2%	2-10%
Platelet count	265×10⁹/L	450×10⁹/L
ESR	65	0-15 mm/hr (M)

**Table 2:** Laboratory investigations. HCV, hepatitis C virus; HBV, hepatitis B virus; HIV, human immunodeficiency virus

Test	Result
Sputum gram stain	Normal study
Sputum AFB stain	Normal study
Sputum fungal stain	Normal study
Sputum GeneXpert	Normal study
Sputum culture	Normal flora
Anti-HCV	Negative
Anti-HIV	Negative
Anti-HBV	Negative

All tests performed on the patient's bronchoalveolar lavage (BAL) fluid, including gram stain, acid-fast bacilli (AFB) stain, fungal stain, GMS stain, fungal culture on Sabouraud agar (after six weeks of incubation), GeneXpert, and malignant cell analysis, returned negative results (Table 3).

**Table 3:** Diagnostic test results for BAL fluid. BAL, bronchoalveolar lavage

Test	Result
BAL fluid gram, AFB and fungal stain	Negative
BAL fluid for GMS stain	Negative
BAL fluid fungal culture (Sabouraud agar)	Negative
BAL fluid GeneXpert	Negative
BAL fluid for malignant cell	Negative

**Figure 1:**
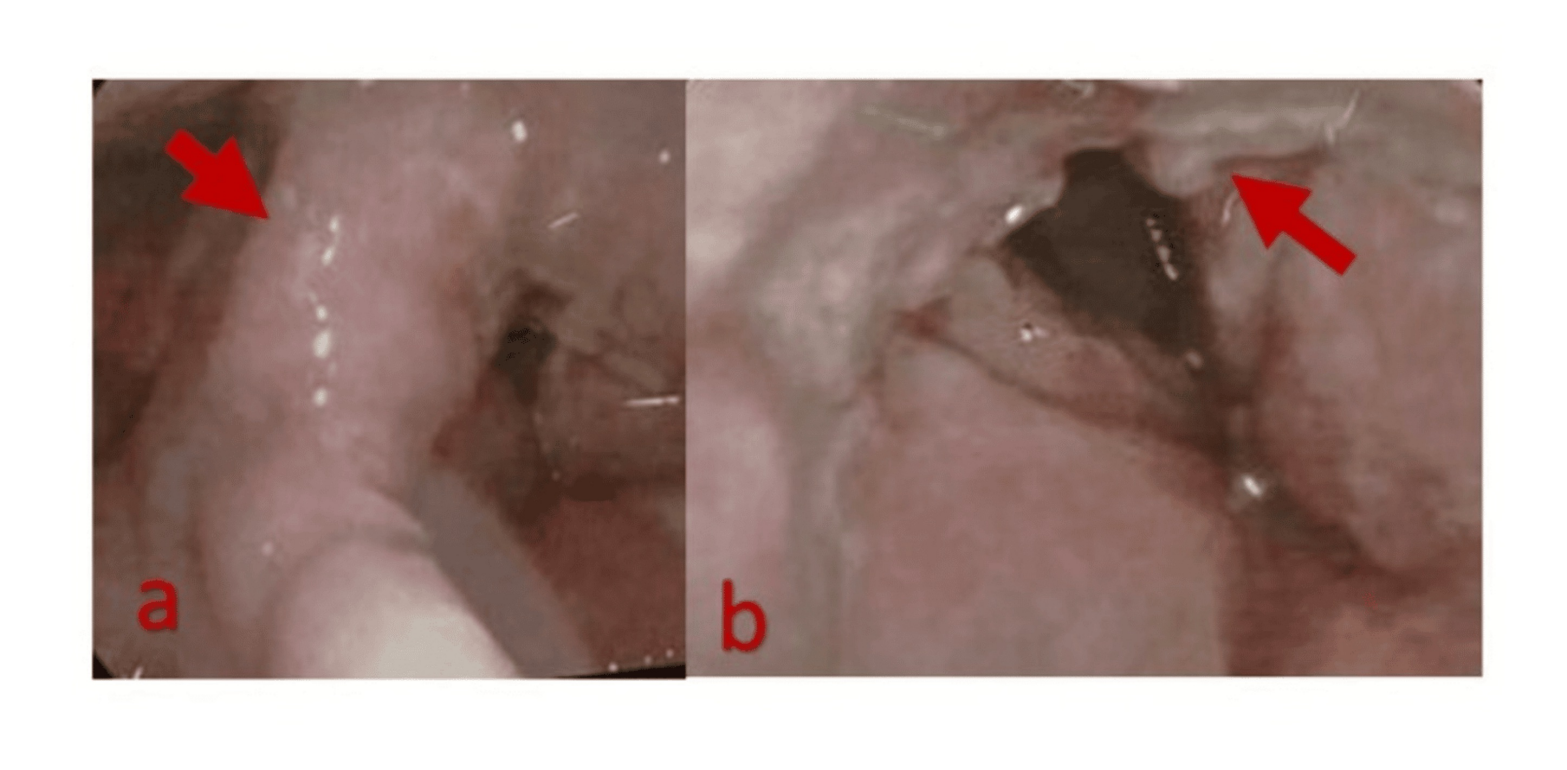
Fiber optic laryngoscopic images show a swollen epiglottis (a) and granulonodularity in the post-arytenoid region (b).

Fiber optic laryngoscopy (FOL) revealed granulonodularity in the posterior larynx and edema of the epiglottis (Figure 1).

**Figure 2:**
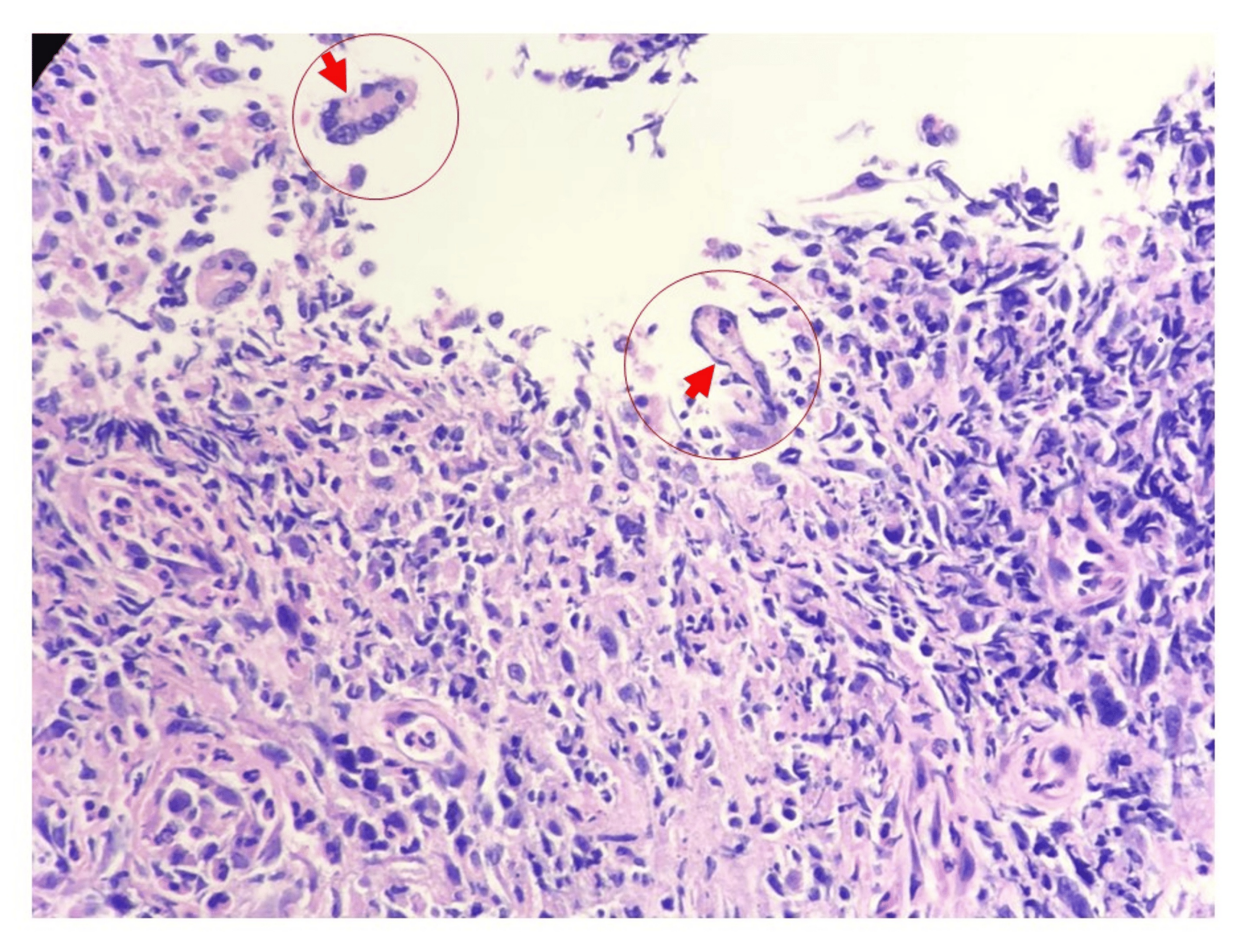
H&E stain shows small, oval intracellular yeast forms within macrophages, highlighted in the circularly marked area.

**Figure 3:**
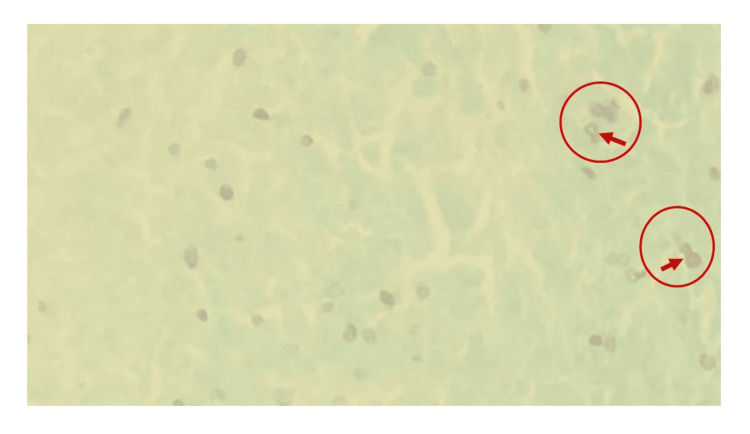
GMS stain shows small, oval yeast forms of Histoplasma with characteristic black staining within macrophages, highlighted in the circularly marked area.

A laryngeal biopsy taken from the soft tissue of both arytenoids revealed greyish-white tissue that was heavily infiltrated with acute and chronic inflammatory cells, along with the presence of multinucleated cells. H&E staining demonstrated spherical to oval intracellular bodies (Figure 2), while GMS stain revealed small, oval yeast forms of Histoplasma with characteristic black staining within macrophages (Figure 3).

The chest CT scan revealed the presence of bullae, fibrotic lesions, focal emphysema, and mild bronchiectasis in both lungs, consistent with sequelae of pulmonary TB. The final diagnosis was primary laryngeal histoplasmosis with post-TB bronchiectasis. The patient was treated with oral itraconazole 200 mg twice daily for one year. Follow-up FOL six months after treatment showed significant improvement, and the patient was advised to continue treatment for a total duration of 12 months for complete recovery.

## Discussion

Histoplasmosis, caused by the dimorphic fungus Histoplasma capsulatum, occurs worldwide and is transmitted via inhalation of spores (microconidia), commonly from environments like bat caves or poultry farms. In the lungs, the fungus transitions from its mycelial form to yeast due to body temperature and is engulfed by macrophages [6]. Mild infections involve limited pulmonary sites and are often asymptomatic, while heavy exposure can lead to severe disease with multiple pulmonary sites, potentially progressing to cavitation or dissemination if left untreated [7].

Histoplasma capsulatum binds to macrophages via HSP60, triggering inflammation and macrophage recruitment. In immunocompetent individuals, the immune system controls or eliminates the fungus. In immunocompromised individuals, it escapes macrophages, proliferates, and spreads, causing disseminated disease [6]. Risk factors for Histoplasma capsulatum infection include smoking, TB history, immunosuppression, endocrine disorders, travel to endemic areas, and occupations like construction, farming, and cave exploration [8].

Histoplasmosis is classified into acute pulmonary (most common), acute disseminated (rare, affecting immunocompromised, elderly, and young children), chronic disseminated (with fever, weight loss, and mucosal ulcers), and chronic pulmonary forms. Laryngeal involvement occurs in 31% of acute pulmonary, 66% of chronic pulmonary, and 19% of acute disseminated histoplasmosis cases. Risk factors include immunosuppression, smoking, TB, endocrine disorders, and travel to endemic areas [9]. Chronic pulmonary histoplasmosis mimics TB with fever, weight loss, and respiratory symptoms. Laryngeal histoplasmosis causes hoarseness, dysphagia, sore throat, and weight loss, with mucosal lesions resembling carcinoma or TB, often part of disseminated disease. Isolated laryngeal histoplasmosis is a very rare condition [10].

We present this case to emphasize the rarity of isolated laryngeal histoplasmosis. Our patient, a smoker with a history of pulmonary TB and newly diagnosed diabetes mellitus, presented with hoarseness but no dyspnea or noisy breathing, despite the severity of the lesion. Examination revealed a swollen arytenoid region, granulonodularity in the posterior larynx, and a tubular, edematous epiglottis, with the glottic region unaffected, likely accounting for the absence of airway obstruction. Previously, two cases of primary laryngeal histoplasmosis were reported in Bangladesh (2012 and 2014). Both involved 60-year-old males with a history of TB, presenting with hoarseness and showing marked improvement after treatment with oral itraconazole and amphotericin B [11].

We have tried to exclude lung involvement by performing a chest CT scan, which showed no pulmonary nodules, consolidations, ground-glass opacities, hilar or mediastinal lymphadenopathy, diffuse reticulonodular infiltrates, calcified pulmonary nodules, broncholiths, or fibrosing mediastinitis, all of which are typical features of histoplasmosis [11]. Additionally, BAL fluid analysis including gram stain, fungal stain, GMS stain, AFB stain, GeneXpert, malignant cell analysis, and fungal culture on Sabouraud agar (after six weeks) were all negative. Furthermore, serum and urine histoplasma antigen testing was unavailable in our country, and the patient declined a lung biopsy.

Furthermore, two additional cases were documented in Colombia and Malaysia [1,8], both involving significant risk factors. The first was a renal transplant patient on immunosuppressive therapy, presenting with hoarseness and supraglottic inflammation. Treatment with intravenous amphotericin B for 10 days followed by oral itraconazole for 12 months led to complete resolution confirmed by flexible laryngoscopy at eight months. The second case involved an immunocompetent individual, a 30-pack-year smoker and pigeon breeder, who presented with hoarseness but no stridor or respiratory distress. This patient received intravenous amphotericin B (0.5 mg/kg/day) for two weeks and oral itraconazole (200 mg three times daily) for two months, achieving full recovery.

The false cords and aryepiglottic folds are the most frequently affected areas in laryngeal histoplasmosis [12]. A biopsy of the laryngeal lesions should be done for culture or histopathological confirmation. Special stains, such as H&E, GMS, and periodic acid-Schiff, highlight narrow-budding yeast within macrophages. Histologically, the disease may resemble TB with tuberculoid granulomas and caseous necrosis [10]. To assess for disseminated disease, chest radiography, bone marrow biopsy, sputum analysis, and biochemical markers are recommended [12].

As per the 2007 update from the Infectious Diseases Society of America, patients with severe progressive disseminated histoplasmosis should first be treated with amphotericin B, followed by itraconazole. For less severe cases, oral itraconazole is recommended. Amphotericin B is typically given at 0.7-1.0 mg/kg, with a total dose of 35 mg/kg over two to four months. The daily dosage of itraconazole is 200 mg [13]. If amphotericin B is not tolerated, alternatives such as oral fluconazole (200-400 mg daily) or ketoconazole (200-400 mg daily) may be used [14]. In our case, the patient was treated with oral itraconazole and showed significant improvement.

## Conclusions

Histoplasmosis should be considered in the differential diagnosis of laryngeal symptoms such as dysphagia and hoarseness, particularly when TB or cancer is initially suspected. The clinical presentation can mimic other common diseases, making diagnosis challenging. A thorough diagnostic workup, including fungal stain, culture, and histopathological examination, is crucial for accurate identification. Early recognition and appropriate antifungal treatment can significantly improve patient outcomes, highlighting the importance of considering this infection in endemic areas.

## References

[1] Moriones Robayo CA, Guerra Ortiz CP (2014). Histoplasmosis laryngeal: report first case in Colombia. Colombia Med.

[2] Siddiqi SH, Stauffer JC (1980). Prevalence of histoplasmin sensitivity in Pakistan. Am J Trop Med Hyg.

[3] Viswanathan R, Chakravarty SC, Randhawa HS, DeMonte AJ (1960). Pilot histoplasmosis survey in Delhi area. Br Med J.

[4] Pervez MM, Cobb B, Matin N, Shahrin L, Ford ER, Pietroni M (2010). Disseminated histoplasmosis in a patient with advanced HIV disease - lessons learnt from Bangladesh. J Health Popul Nutr.

[5] Teoh JW, Hassan F, Mohamad Yunus MR (2013). Laryngeal histoplasmosis: an occupational hazard. Singapore Med J.

[6] Ansari HA, Saeed N, Khan N, Hasan N (2016). Laryngeal histoplasmosis. BMJ Case Rep.

[7] Wolf J, Blumberg HM, Leonard MK (2004). Laryngeal histoplasmosis. Am J Med Sci.

[8] Sataloff RT, Wilborn A, Prestipino A, Hawkshaw M, Heuer RJ, Cohn J (1993). Histoplasmosis of the larynx. Am J Otolaryngol.

[9] Bist SS, Sandhirr S, Shirazi N, Agarwal V, Bharti B (2015). Primary histoplasmosis of larynx mimicking as laryngeal carcinoma. Int J Phonosurg Laryngol.

[10] Pochini Sobrinho F, Della Negra M, Queiroz W, José Ribeiro U, Bittencourt S, Burlamaqui Klautau G (2007). Histoplasmosis of the larynx. Braz J Otorhinolaryngol.

[11] Rahim MA, Zaman S, Amin MR, Uddin KN, Ma JC (2020). Histoplasmosis: an emerging or neglected disease in Bangladesh? A systematic review. Oman Med J.

[12] Sonkhya N, Mehta R, Sonkhya D, Gupta S, Faujdar M (2013). Primary histoplasmosis of larynx: a case series and review of literature. Int J Otolaryngol Head Neck Surg.

[13] Wheat LJ, Freifeld AG, Kleiman MB, Baddley JW, McKinsey DS, Loyd JE, Kauffman CA (2007). Clinical practice guidelines for the management of patients with histoplasmosis: 2007 update by the Infectious Diseases Society of America. Clin Infect Dis.

[14] Sharma P, Singh G, Sharma D, Giri S (2020). Laryngeal histoplasmosis mimicking glottic cancer in an immunocompetent host. Int J Clin Diagn Pathol.

